# The Ultrabroad-Spectrum Beta-Lactamase Inhibitor QPX7728 Restores the Potency of Multiple Oral Beta-Lactam Antibiotics against Beta-Lactamase-Producing Strains of Resistant *Enterobacterales*

**DOI:** 10.1128/aac.02168-21

**Published:** 2022-02-15

**Authors:** Olga Lomovskaya, Debora Rubio-Aparicio, Ruslan Tsivkovski, Jeff Loutit, Michael Dudley

**Affiliations:** a Qpex Biopharma, Inc., San Diego, California, USA

**Keywords:** QPX7728, oral beta-lactams, serine beta-lactamases, metallo-beta-lactamases, *Enterobacterales*, beta-lactamase inhibitor

## Abstract

QPX7728 is a cyclic boronate ultrabroad-spectrum beta-lactamase inhibitor, with potent activity against both serine beta-lactamases and metallo-beta-lactamases. QPX7728 can be delivered systemically by the intravenous (i.v.) or oral route of administration. Oral beta-lactam antibiotics alone or in combination with QPX7728 were evaluated for (i) sensitivity to hydrolysis by various common beta-lactamases and inhibition of hydrolysis by QPX7728, (ii) the impact of non-beta-lactamase-mediated resistance mechanisms on potency of beta-lactams, and (iii) *in vitro* activity against a panel of clinical strains producing diverse beta-lactamases. The carbapenem tebipenem had stability for many serine beta-lactamases from all molecular classes, followed by the cephalosporin ceftibuten. Addition of QPX7728 to tebipenem, ceftibuten, and amdinocillin completely reversed beta-lactamase-mediated resistance in cloned beta-lactamases from serine enzyme and metalloenzyme classes; the degree of potentiation of other beta-lactams varied according to the beta-lactamase produced. Tebipenem, ceftibuten, and cefixime had the lowest MICs against laboratory strains with various combinations of beta-lactamases and the intrinsic drug resistance mechanisms of porin and efflux mutations. There was a high degree of correlation between potency of various combinations against cloned beta-lactamases and efflux/porin mutants and the activity against clinical isolates, showing the importance of inhibition of beta-lactamase along with minimal impact of general intrinsic resistance mechanisms affecting the beta-lactam. Tebipenem and ceftibuten appeared to be the best beta-lactam antibiotics when combined with QPX7728 for activity against *Enterobacterales* that produce serine beta-lactamases or metallo-beta-lactamases.

## INTRODUCTION

There remains a significant unmet need for new oral and intravenous (i.v.) agents to treat Gram-negative infections because of resistant bacteria in both the community and hospital settings. Some progress has been made in the successful development of new agents to address drug-resistant infections, particularly i.v. agents, for resistant Gram-negative bacteria (1). While some of these i.v. agents have addressed urgent antimicrobial resistance threats like carbapenem-resistant *Enterobacterales* (CRE), their activity is largely confined to strains producing the Klebsiella pneumoniae carbapenemase (KPC) beta-lactamase; none of the recently regulatory agency-approved agents have reliable activity against metallo-beta-lactamase (MBL)-producing CRE (1). Notably, no new oral agents to treat extended-spectrum beta-lactamase (ESBL)-producing or fluoroquinolone-resistant *Enterobacterales* or CRE have been introduced into clinical use.

Surveillance studies have demonstrated a steady increase in recovery of drug-resistant Gram-negative pathogens in clinical settings outside of acute care hospitals (2, 3). In a 2017 survey of 1,831 Escherichia coli urinary tract isolates in the United States from both hospital- and community-acquired infections, the proportion expressing an ESBL phenotype was 15.7%, with 24.3% and 32.1% of isolates resistant to levofloxacin and trimethoprim-sulfamethoxazole, respectively (4).

As was previously observed with ESBLs, CRE have spread from the inpatient setting to long-term institutional care centers and ultimately to the outpatient in community settings. Several recent reports identified proportions ranging from 0.04% to 29.5% of either community-associated or community-onset CRE among studied samples (5). Importantly, outpatient care facilities can play a role in the transmission of both serine beta-lactamase- and metallo-beta-lactamase-producing isolates (6–8).

Inhibition of beta-lactamases with small-molecule inhibitors (beta-lactamase inhibitors [BLIs]) is a broadly recognized strategy to overcome class-specific resistance by preventing hydrolysis and inactivation of beta-lactams and restore their potency against Gram-negative pathogens (9, 10). Since 2015, three new beta-lactamase inhibitors, avibactam, vaborbactam, and relebactam, have been introduced into clinical practice (11). All of them have a broader profile of beta-lactamase inhibition, notably including KPC and class C beta-lactamases, than clavulanic acid, tazobactam, and sulbactam. Unfortunately, none of these new BLIs inhibit metallo-beta-lactamases, whose incidence is increasing across various species worldwide (9, 10). None of the newer BLIs show oral bioavailability.

**FIG 1:**
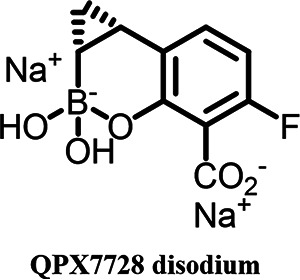
Chemical structure of QPX7728.

QPX7728 (Fig. 1) is a member of a class of cyclic boronates that gave rise to vaborbactam (12, 13), the first boronic BLI introduced into clinical use. Ongoing innovation with this new class resulted in QPX7728, an ultrabroad-spectrum beta-lactamase inhibitor with potent activity against both serine beta-lactamases and metallo-beta-lactamases (14–17). QPX7728 is minimally affected by efflux in either *Enterobacterales* or in Pseudomonas aeruginosa (18), and changes in porins affect QPX7728 to a lesser degree than vaborbactam (18). QPX7728 can be delivered by the i.v. or oral route of administration. Given its broad spectrum of beta-lactamase inhibition, which includes enzymes that degrade oral carbapenems, cephalosporins, and penicillins, we evaluated multiple oral beta-lactam antibiotics alone or in combination with QPX7728. The aim of these studies was to assess the properties of various combinations for (i) hydrolysis of beta-lactams by common beta-lactamases and QPX7728 reversal of resistance, (ii) impact of non-beta-lactamase-mediated general intrinsic resistance mechanisms, and (iii) activity of combinations with QPX7728 against clinical isolates producing diverse beta-lactamases.

## RESULTS

### Oral beta-lactams differ in potency against beta-lactamase-producing strains.

The set of isogenic strains of Klebsiella pneumoniae expressing individual cloned beta-lactamases was used to evaluate susceptibility of beta-lactams to beta-lactamase-mediated hydrolysis (14). The potency of the antibiotics varied widely, indicating different susceptibilities to beta-lactamase-mediated hydrolysis (Table 1). The carbapenem tebipenem had the lowest MICs, with higher values observed for carbapenemase-producing strains. Ceftibuten had the lowest MIC against producers of class A ESBLs, notably CTX-M-14/CTX-M-15, SHV-5/SHV-12, and KPC-2. Ceftibuten MICs against the MBL-producing strains were also lower than those of other cephalosporins. Cefditoren, cefixime, cefdinir, and cefpodoxime had relatively low MICs against many beta-lactamase producers. Cefaclor, cefuroxime, and cephalexin were the least potent compounds. Amdinocillin was less susceptible to hydrolysis by various class C enzymes than were cephalosporins but was poorly active against KPC-, metallocarbapenemase-, and ESBL-producing strains (Table 2).

**TABLE 1:** *In vitro* potencies of oral beta-lactams in combination with QPX7728 at 4 μg/mL against isogenic strains of Klebsiella pneumoniae expressing cloned beta-lactamases^*a*^

Strain	Beta-lactamase	MIC (μg/mL)											
		Tebipenem	Tebipenem + QPX7728	Ceftibuten	Ceftibuten + QPX7728	Cefixime	Cefixime + QPX7728	Cefditoren	Cefditoren + QPX7728	Cefdinir	Cefdinir + QPX7728	Cefpodoxime	Cefpodoxime + QPX7728
KPM1116	None	≤0.06	≤0.06	≤0.06	≤0.06	≤0.06	≤0.06	0.25	≤0.06	0.125	≤0.06	≤0.06	≤0.06
KPM1113	KPC-2	0.5	≤0.06	0.5	≤0.06	2	≤0.06	>64	≤0.06	16	≤0.06	16	≤0.06
KPM1031	CTX-M-14	≤0.06	≤0.06	2	≤0.06	16	≤0.06	32	≤0.06	256	≤0.06	256	≤0.06
KPM1114	CTX-M-15	≤0.06	≤0.06	4	≤0.06	32	≤0.06	64	≤0.06	256	≤0.06	>256	≤0.06
KPM1115	SHV-12	≤0.06	≤0.06	2	≤0.06	16	≤0.06	32	≤0.06	4	≤0.06	16	≤0.06
KPM3258	SHV-5	≤0.06	≤0.06	2	≤0.06	16	≤0.06	64	≤0.06	8	≤0.06	64	≤0.06
KPM1112	TEM-10	≤0.06	≤0.06	0.25	≤0.06	4	≤0.06	16	≤0.06	0.5	≤0.06	8	≤0.06
KPM3736	PER-2	≤0.06	≤0.06	16	≤0.06	16	≤0.06	16	≤0.06	32	≤0.06	64	0.5 **(8)**
KPM3812	VEB-2	≤0.06	≤0.06	16	≤0.06	64	≤0.06	64	≤0.06	32	≤0.06	16	≤0.06
KPM1956	P99-like AmpC	≤0.06	≤0.06	64	≤0.06	128	≤0.06	64	≤0.06	64	≤0.06	32	≤0.06
KPM1030	DHA-1	≤0.06	≤0.06	16	≤0.06	32	≤0.06	4	≤0.06	16	≤0.06	16	≤0.06
KPM1045	CMY-2	≤0.06	≤0.06	32	≤0.06	>64	≤0.06	64	≤0.06	32	≤0.06	128	≤0.06
KPM3352	MIR-1	≤0.06	≤0.06	64	≤0.06	>64	≤0.06	64	≤0.06	64	≤0.06	>64	2 **(32)**
KPM1939	OXA-48	0.5	≤0.06	≤0.06	≤0.06	≤0.06	≤0.06	1	≤0.06	16	≤0.06	≤0.06	≤0.06
KPM1932	NDM-1	32	≤0.06	64	0.125	256	0.5 **(8)**	256	0.25 **(4)**	256	1 **(16)**	256	2 **(32)**
KPM1935	VIM-1	8	≤0.06	32	≤0.06	256	≤0.06	256	≤0.06	256	0.125	256	0.125
KPM1996	IMP-1	4	≤0.06	64	≤0.06	128	≤0.06	128	≤0.06	64	0.25	128	0.25 **(4)**

a Bold numbers in parentheses indicate MIC ratios between the beta-lactamase-producing strains and the vector-alone strain that are equal to or higher than 4. Data for ceftibuten, tebipenem, and cefpodoxime were presented earlier (14).

**TABLE 2:** *In vitro* potencies of additional oral beta-lactams in combination with QPX7728 at 4 μg/mL against isogenic strains of Klebsiella pneumoniae expressing cloned beta-lactamases^*a*^

Strain	Beta-lactamase	MIC (μg/mL)							
		Amdinocillin	Amdinocillin + QPX7728	Cefaclor	Cefaclor + QPX7728	Cefuroxime	Cefuroxime + QPX7728	Cephalexin	Cephalexin + QPX7728
KPM1116	None	0.25	0.25	0.5	0.25	2	0.5	4	2
KPM1113	KPC-2	64	0.25	>64	0.25	64	0.5	128	2
KPM1031	CTX-M-14	0.5	0.125	256	0.125	>256	0.5	256	2
KPM1114	CTX-M-15	1	0.25	256	0.25	>256	1	256	2
KPM1115	SHV-12	8	0.25	256	0.25	32	0.5	64	2
KPM3258	SHV-5	16	0.25	128	0.25	64	1	64	8
KPM1112	TEM-10	64	0.25	4	0.25	4	1	8	4
KPM3736	PER-2	64	0.25	128	0.25	>64	1	256	4
KPM3812	VEB-2	16	0.25	64	0.25	64	0.5	64	2
KPM1956	P99-like AmpC	0.5	0.25	>256	0.5	128	1	>256	4
KPM1030	DHA-1	0.25	0.25	64	0.25	32	2	>64	4
KPM1045	CMY-2	8	0.25	256	0.25	32	1	128	2
KPM3352	MIR-1	1	0.5	>64	0.5	>64	1	>64	4
KPM1939	OXA-48	0.5	0.25	32	0.25	4	1	8	2
KPM1932	NDM-1	8	0.25	>256	8 (**32)**	>256	32 **(64)**	>256	32 **(16)**
KPM1935	VIM-1	>256	0.5	>256	0.5	>256	2 **(4)**	256	4
KPM1996	IMP-1	16	0.25	128	1 **(4)**	256	2 **(4)**	128	8 **(4)**

a Bold numbers in parentheses indicate MIC ratios between the beta-lactamase-producing strains and the vector-alone strain that are equal to or higher than 4.

### Enhancement of potency of some, but not all, oral beta-lactams by QPX7728 varies according to beta-lactamase.

Evaluation of the panel of cloned beta-lactamase-producing strains demonstrated that the potencies of tebipenem, ceftibuten, and amdinocillin were significantly enhanced by QPX7728 irrespective of a beta-lactamase present. The ratios of antibiotic MICs with QPX7728 for beta-lactamase-producing strains versus the empty-vector-alone-producing strain KPM1113 was 2 or less, indicating complete inhibition of various beta-lactamases (Table 1). For all other tested antibiotics, the extent of potentiation by QPX7728 depended on the beta-lactamase produced and the intrinsic stability of the beta-lactam to the enzyme. The MICs of cefditoren/QPX7728, cefixime/QPX7728, cefdinir/QPX7728, and cefaclor/QPX7728 were increased 4-fold or more over those for the vector-alone strain against the strains producing MBLs, particularly NDM-1. Higher antibiotic/QPX7728 MICs against the MBL-producing strains were observed for other beta-lactam combinations as well. In addition, the potency of cefpodoxime/QPX72728 and cefuroxime/QPX7728 combinations was reduced against the strains producing class C MIR-1 and DHA-1 beta-lactamases, respectively. Cefpodoxime/QPX7728 and cephalexin/QPX7728 also had reduced potencies against the strains producing class A ESBLs PER-2 and SHV-12 (Tables 1 and 2).

### Efflux and permeability mutations have variable effects on each beta-lactam antibiotic and can limit the impact of beta-lactamase inhibition by QPX7728.

When beta-lactamases are completely inhibited by BLIs, it is non-beta-lactamase-mediated general intrinsic resistance mechanisms of efflux and porin mutations that determine the potencies of beta-lactam/BLI combinations. To characterize differences, the *in vitro* potencies of various beta-lactams were assessed against the set of isogenic strains of K. pneumoniae with various combinations of efflux and porin mutations (18).

The beta-lactams differed in intrinsic potency as judged from MICs against strain KPM 1026A, a derivative of a genetically tractable, prototypical strain, ATCC 43816, with wild-type susceptibility (Table 3). The carbapenem tebipenem (MIC ≤ 0.06 μg/ml) was the most potent strain, with ceftibuten, cefixime, cefdinir, and cefpodoxime having similar levels of potency (MIC = 0.125 μg/mL), followed by cefditoren, cefaclor, and amdinocillin (MIC = 0.5 μg/mL). Cefuroxime and cephalexin were the least potent agents, with MICs of 2 to 4 μg/mL.

**TABLE 3:** Effects of efflux and porins on activity of beta-lactam antibiotics against a panel of isogenic strains of Klebsiella pneumoniae with efflux and porin mutations^*a*^

Strain	Genotype	AcrAB-TolC	OmpK35	OmpK36	MIC (μg/mL)									
					Tebipenem	Ceftibuten	Cefixime	Cefpodoxime	Cefdinir	Cefditoren	Cefaclor	Mecillinam	Cefuroxime	Cephalexin
KPM1026A^*b*^	WT	WT	WT	WT	≤0.06	0.125	0.125	0.125	0.125	0.5	0.5	0.5	2	4
PAM2696	Δ*acrAB*	None	WT	WT	≤0.06	≤0.06	≤0.06	≤0.06	≤0.06	≤0.06	1	≤0.06	0.125	4
KPM1027^*c*^	*ramR* ^*f*^	Up	Down	WT	≤0.06	0.25	0.5	2	1	2	1	2	32	8
KPM2600	Δ*ompK35*	WT	NF	WT	≤0.06	0.125	0.125	0.25	0.125	0.25	0.5	0.5	4	4
KPM2610	*ramR*^*f*^ Δ*ompK35*	Up	NF	WT	≤0.06	0.25	0.5	2	2	2	1	2	16	8
KPM2592	Δ*ompK36*	WT	WT	NF	0.125	0.25	0.25	0.5	1	0.5	2	>64	8	16
KPM2040^*d*^	*ompK36_2067*	WT	WT	NF	0.125	0.25	0.25	0.5	2	1	2	16	16	16
KPM2613	*ompK36_2067* Δ*ompK35*	WT	NF	NF	0.5	0.25	0.5	1	2	1	4	>64	16	32
KPM2126	*ramR*^*e*^ *ompK36_2013*	Up	Down	NF	1	1	2	4	8	4	16	>64	64	>64
KPM2658	*ramR*^*f*^ Δ*ompK36*	Up	Down	NF	2	1	1	4	8	4	16	>64	32	>64

a All strains contain chromosomal SHV enzyme, encoded by *bla*_SHV-24_. WT, wild type; NF, nonfunctional; Up, upregulated; Down, downregulated.

b KPM1026a is a streptomycin-resistant mutant of the wild-type strain KPM1001 (ATCC 43816). It contains a functional *acrAB* operon and functional genes *ompK35* and *ompK36*.

c KPM1027 is a derivative of KPM1026a selected on tigecycline. It has an inactivating mutation in the negative regulator gene *ramR* (frameshift from amino acid 46 in the RamR protein) and as a result has the *acrAB* operon overexpressed ∼3-fold and the *ompK35* gene downregulated ∼10-fold relative to KPM1026a. Expression of *ompK36* in KPM1027 is unchanged relative to KPM1026a.

d Insertion of base A at nucleotide 160 of *ompK*36, causing a frameshift from amino acid 54 of OmpK36.

e C364T substitution in *ramR* created TAG at amino acid 122, resulting in overexpression of *acrAB* and downregulation of *ompK35*.

f Insertion of 8 bp in *ramR* causing a frameshift from amino acid 46, resulting in overexpression of *acrAB* and downregulation of *ompK35*.

Deletion of the major efflux pump AcrAB-TolC (KPM2696) increased the potencies of most beta-lactams (2- to 16-fold). Interestingly, the MICs of cefaclor and cephalexin were not affected by pump inactivation, suggesting that these agents are poor substrates for this pump (Table 3).

Inactivation of the porin OmpK35 alone had minimal effects on the potency of the oral beta-lactams tested (see KPM1026a versus KPM2600 in Table 3). Inactivation of OmpK36 decreased the potency of beta-lactams 4- to 32-fold (see KPM1026a versus KPM2592 and KPM2040). Because of the different intrinsic potencies, which ranged from ≤0.06 to 0.125 μg/mL for tebipenem, ceftibuten, cefdinir, cefpodoxime, and cefixime to 2 to 4 μg/mL for cefuroxime and cephalexin, the MICs for OmpK36 mutants amplified these differences and ranged from 0.125 to 0.25 μg/mL to 8 to 16 μg/mL. Inactivation of both porins decreased the potencies of some beta-lactams (tebipenem, cefixime, cefpodoxime, cefaclor, cephalexin, and amdinocillin) an additional 2- to 4-fold compared to inactivation of OmpK36 alone (compare KPM2040 versus KPM2613). As a result, MIC of double-porin mutants ranged from 0.25 to 0.5 μg/mL for ceftibuten, tebipenem, and cefixime to 16 to >64 μg/mL for cefuroxime, cephalexin, and amdinocillin (Table 3).

Increased efflux by the AcrAB-TolC efflux pump did not appear to affect the potency of tebipenem but had a 2- to 16-fold effect on the potency of other beta-lactams (see KPM1026a versus KPM1027). The MIC of efflux overexpressing mutants ranged from ≤0.06 to 0.25 μg/mL for tebipenem and ceftibuten to 16 to 32 μg/mL for cefuroxime and cephalexin.

The highest MICs were recorded for the strains KPM2126 and KPM2658, which had AcrAB overexpressed, OmpK35 downregulated, and OmpK36 inactivated. Based on the potency against these strains, the evaluated beta-lactams were ranked in the following order: ceftibuten (MIC, 1 μg/mL) > tebipenem = cefixime (MIC, 1 to 2 μg/mL) > cefditoren = cefpodoxime (MIC, 4 μg/mL) > cefdinir (MIC, 8 μg/mL) > cefaclor (MIC, 16 μg/mL) > cefuroxime (MIC, 32 to 64 μg/mL) > cephalexin = amdinocillin (MIC, >64 μg/mL) (Table 3).

### QPX7728 enhanced the potencies of oral beta-lactams against the panel of beta-lactamase-producing clinical isolates of *Enterobacterales*.

A total of 230 clinical isolates of *Enterobacterales* from the Qpex internal collection were selected to represent a broad cross-section of organisms, genotypes, and beta-lactamases, including carbapenemase-producing strains (see Table S1 in the supplemental material). Evaluation of beta-lactam/QPX7728 combinations against this panel demonstrated that QPX7728 significantly enhanced the potencies of several oral beta-lactams. When tebipenem, ceftibuten, cefixime, cefditoren, cefdinir, and cefpodoxime were tested alone, MIC_50_s ranged from 4 μg/mL to >64 μg/mL and MIC_90_s were >64 μg/mL. MIC_50_s and MIC_90_s of QPX7728 combinations ranged from ≤0.06 μg/mL to 1 μg/mL and from 0.5 μg/mL to 8 μg/mL, respectively (Table 4). Thus, MIC_50_s and MIC_90_s for these beta-lactams were reduced 64- to >128-fold and 8- to >128-fold, respectively, when they were combined with QPX7728. QPX7728 had little or no effect on the potencies of amdinocillin, cefuroxime, cefaclor, and cephalexin, with MIC_50_s affected 4- to >64-fold and no effect on MIC_90_s (Table 5).

**TABLE 4:** *In vitro* potencies of oral beta-lactams in combination with QPX7728 at 4 μg/mL or comparator combinations against beta-lactamase-producing strains of *Enterobacterales* according to beta-lactamase

Group and MIC parameter	Value (μg/mL)											
	Tebipenem	Tebipenem + QPX7728	Ceftibuten	Ceftibuten + QPX7728	Cefixime	Cefixime + QPX7728	Cefditoren	Cefditoren + QPX7728	Cefdinir	Cefdinir + QPX7728	Cefpodoxime	Cefpodoxime + QPX7728
All (*n* = 230)												
MIC_50_	4	≤0.06	32	0.25	>64	0.5	>64	0.5	>64	0.5	>64	1
MIC_90_	>64	0.5	>64	2	>64	4	>64	4	>64	8	>64	8
Range	≤0.06 to >64	≤0.06 to >64	0.125 to >64	≤0.06 to 4	0.25 to >64	≤0.06 to >64	0.5 to >64	≤0.06 to >64	0.25 to >64	≤0.06 to >64	1 to >64	≤0.06 to >64
ESBL (*n* = 84)^*a*^												
MIC_50_	≤0.06	≤0.06	8	0.125	>64	0.125	>64	0.25	>64	0.25	>64	0.5
MIC_90_	2	0.5	>64	1	>64	1	>64	2	>64	4	>64	4
Range	≤0.06 to 16	≤0.06 to 1	0.125 to >64	≤0.06 to 2	0.5 to >64	≤0.06 to 16	0.5 to >64	≤0.06 to >64	0.25 to >64	≤0.06 to 16	1 to >64	≤0.06 to 16
Plasmidic class C (*n* = 12)^*b*^												
MIC_50_	≤0.06	≤0.06	>64	0.5	>64	0.5	>64	1	>64	2	>64	1
MIC_90_	2	0.25	>64	2	>64	4	>64	4	>64	8	>64	8
Range	≤0.06 to 2	≤0.06 to 1	64 to >64	0.125 to 2	>64	≤0.06 to 4	32 to >64	0.25 to 4	64 to >64	0.25 to 16	>64	0.25 to 8
KPC (*n* = 48)^*c*^												
MIC_50_	32	≤0.06	16	0.25	>64	0.5	>64	1	>64	1	>64	2
MIC_90_	>64	2	>64	2	>64	4	>64	32	>64	8	>64	8
Range	0.125 to >64	≤0.06 to 32	0.125 to >64	≤0.06 to 2	0.25 to >64	≤0.06 to >64	1 to >64	≤0.06 to >64	8 to >64	≤0.06 to >64	1 to >64	≤0.06 to >64
OXA-48 (*n* = 48)												
MIC_50_	32	0.5	32	0.25	>64	0.5	>64	1	>64	2	>64	2
MIC_90_	64	0.5	>64	1	>64	2	>64	4	>64	8	>64	4
Range	≤0.06 to >64	≤0.06 to 1	0.25 to >64	≤0.06 to 2	0.25 to >64	≤0.06 to 64	16 to >64	0.125 to 32	64 to >64	≤0.06 to >64	2 to >64	0.125 to 16
MBL (*n* = 36)												
MIC_50_	32	≤0.06	>64	1	>64	2	>64	0.5	>64	0.5	>64	8
MIC_90_	>64	0.25	>64	2	>64	32	>64	4	>64	>64	>64	32
Range	≤0.06 to >64	≤0.06 to 16	0.5 to >64	≤0.06 to 4	0.5 to >64	≤0.06 to 64	1 to >64	≤0.06 to >64	0.5 to >64	≤0.06 to >64	1 to >64	≤0.06 to 64

a ESBL, no coproduced carbapenemases or plasmidic class C enzymes.

b Plasmidic class C, no carbapenemases.

c Many carbapenemase-producing strains also coproduced ESBLs or class C beta-lactamases.

**TABLE 5:** *In vitro* potencies of additional oral beta-lactams in combination with QPX7728 at 4 μg/mL or comparator combinations against beta-lactamase-producing strains of *Enterobacterales* according to beta-lactamase

Group and MIC parameter	Value (μg/mL)											
	Amdinocillin	Amdinocillin + QPX7728	Cefaclor	Cefaclor + QPX7728	Cefuroxime	Cefuroxime + QPX7728	Cephalexin	Cephalexin + QPX7728	Meropenem	Meropenem + QPX7728	Ceftazidime	Ceftazidime + avibactam
All (*n* = 230)												
MIC_50_	>64	1	>64	4	>64	32	>64	32	4	≤0.06	>64	1
MIC_90_	>64	>64	>64	64	>64	>64	>64	>64	>64	0.25	>64	>64
Range	0.5 to >64	0.125 to >64	2 to >64	≤0.06 to >64	8 to >64	0.5 to >64	16 to >64	1 to >64	≤0.06 to >64	≤0.06 to 4	0.25 to >64	≤0.06 to >64
ESBL (*n* = 84)^*a*^												
MIC_50_	>64	1	>64	2	>64	8	>64	8	≤0.06	≤0.06	64	0.5
MIC_90_	>64	8	>64	16	>64	64	>64	>64	4	0.25	>64	4
Range	0.5 to >64	0.125 to >64	2 to >64	≤0.06 to >64	8 to >64	0.5 to >64	16 to >64	1 to >64	≤0.06 to 16	≤0.06 to 0.5	0.25 to >64	≤0.06 to 64
Plasmidic class C (*n* = 12)^*b*^												
MIC_50_	>64	1	>64	2	>64	32	>64	32	≤0.06	≤0.06	>64	0.5
MIC_90_	>64	>64	>64	32	>64	>64	>64	>64	2	0.125	>64	4
Range	0.5 to >64	0.5 to >64	>64	1 to 32	32 to >64	8 to >64	>64	8 to >64	≤0.06 to 4	≤0.06 to 0.5	2 to >64	0.25 to 16
KPC (*n* = 48)^*c*^												
MIC_50_	>64	2	>64	8	>64	32	>64	32	16	≤0.06	>64	2
MIC_90_	>64	>64	>64	>64	>64	>64	>64	>64	>64	1	>64	8
Range	>64	0.25 to >64	64 to >64	0.25 to >64	8 to >64	1 to >64	64 to >64	2 to >64	0.5 to >64	≤0.06 to 4	0.5 to >64	0.125 to >64
OXA-48 (*n* = 48)												
MIC_50_	>64	1	>64	8	>64	32	>64	32	16	0.125	>64	2
MIC_90_	>64	16	>64	>64	>64	>64	>64	>64	64	0.25	>64	4
Range	0.5 to >64	0.25 to >64	>64	0.5 to >64	32 to >64	1 to >64	>64	2 to >64	≤0.06 to 64	≤0.06 to 0.25	0.5 to >64	0.25 to 64
MBL (*n* = 36)												
MIC_50_	>64	1	>64	16	>64	>64	>64	64	16	≤0.06	>64	>64
MIC_90_	>64	>64	>64	>64	>64	>64	>64	>64	>64	0.125	>64	>64
Range	0.5 to >64	0.25 to >64	2 to >64	0.25 to >64	8 to >64	0.5 to >64	16 to >64	2 to >64	≤0.06 to >64	≤0.06 to 2	2 to >64	≤0.06 to >64

a ESBL, no coproduced carbapenemases or plasmidic class C enzymes.

b Plasmidic class C, no carbapenemases.

c Many carbapenemase-producing strains also coproduced ESBLs or class C beta-lactamases.

### Enhancement of beta-lactam potency by QPX7728 was observed across all enzyme subgroups.

**FIG 2:**
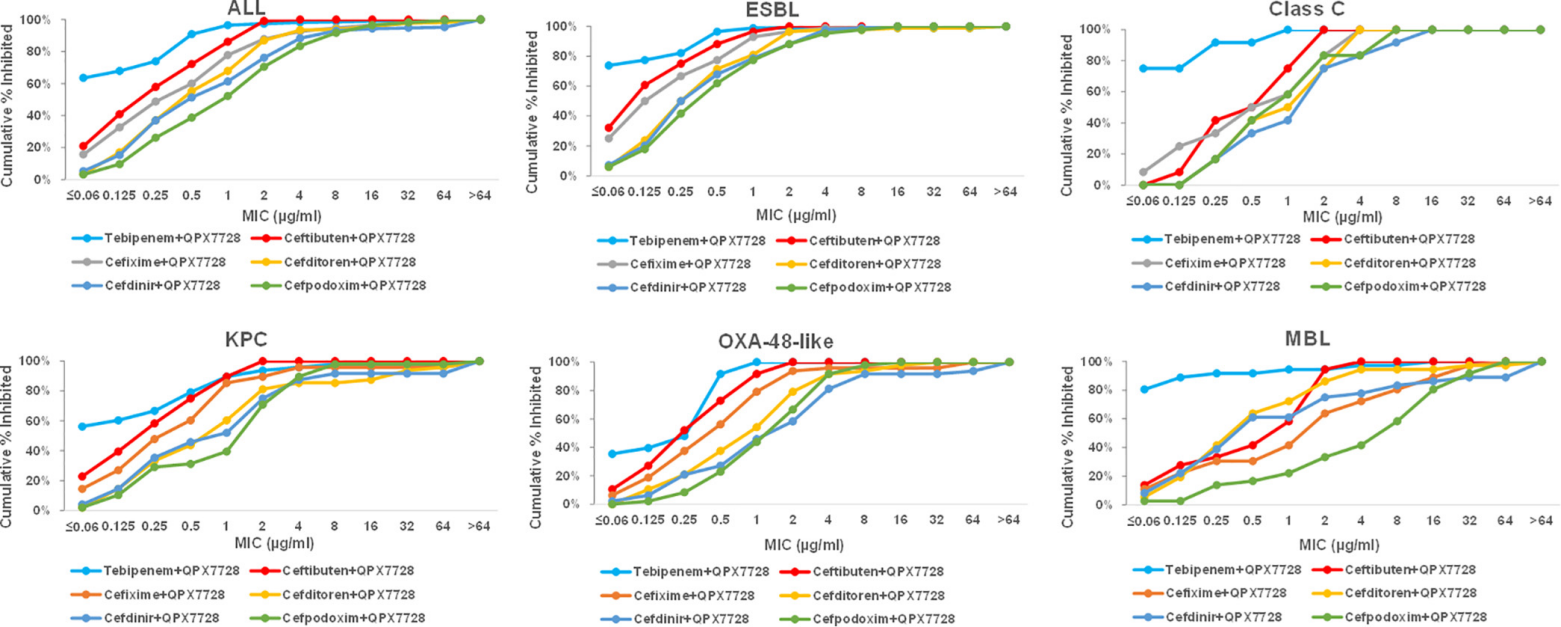
MIC distribution of QPX7728 combinations with several oral beta-lactams according to beta-lactamase. QPX7728 was tested at 4 μg/mL.

QPX7728 enhanced the potency of oral beta-lactams across all enzyme subgroups of the panel, including MBL-producing strains (Tables 4 and 5 and Fig. 2). QPX7728 combined with tebipenem was the most potent overall, with MIC_90_s ranging from 0.25 μg/mL to 2 μg/mL. MIC_90_s of tebipenem/QPX7728 were similar for ESBL OXA-48-, plasmidic class C-, and MBL-producing organisms (0.25 to 0.5 μg/mL); the lowest potency (MIC_90_ of 2 μg/mL) was observed in KPC-producing strains. QPX7728/ceftibuten was the next potent combination, with similar potencies against all enzyme subgroups (MIC_90_s of 1 to 2 μg/mL).

Cefditoren and cefixime combinations appeared to be similar in overall potency, with MIC_90_ of 4 μg/mL. QPX7728 combined with cefditoren had the same potency against many serine beta-lactamases and metallo-beta-lactamases (MIC_90_ of 4 μg/mL); its potency appeared to be reduced against the KPC-producing strains (MIC_90_ of 32 μg/mL). MIC_90_ of QPX7728 in combination with cefixime was in the 1- to 4-μg/mL range against the strains producing serine beta-lactamases but was 32 μg/mL for the MBL-producing strains. A similar trend was observed for QPX7728 in combination with cefdinir and cefpodoxime except that these combinations appeared to be less potent overall (MIC_90_s for serine beta-lactamases of 4 to 8 μg/mL) (Table 4 and Fig. 2).

Combinations of QPX7728 with amdinocillin, cefaclor, cefuroxime, and cephalexin had low potencies, with MIC_90_s of 8 to >64 μg/mL against all groups (Table 4).

### The *in vitro* potencies of various oral beta-lactam/QPX7728 combinations are determined by both the inhibitory activity of QPX7728 and the impact of general intrinsic/MDR mechanisms.

**FIG 3:**
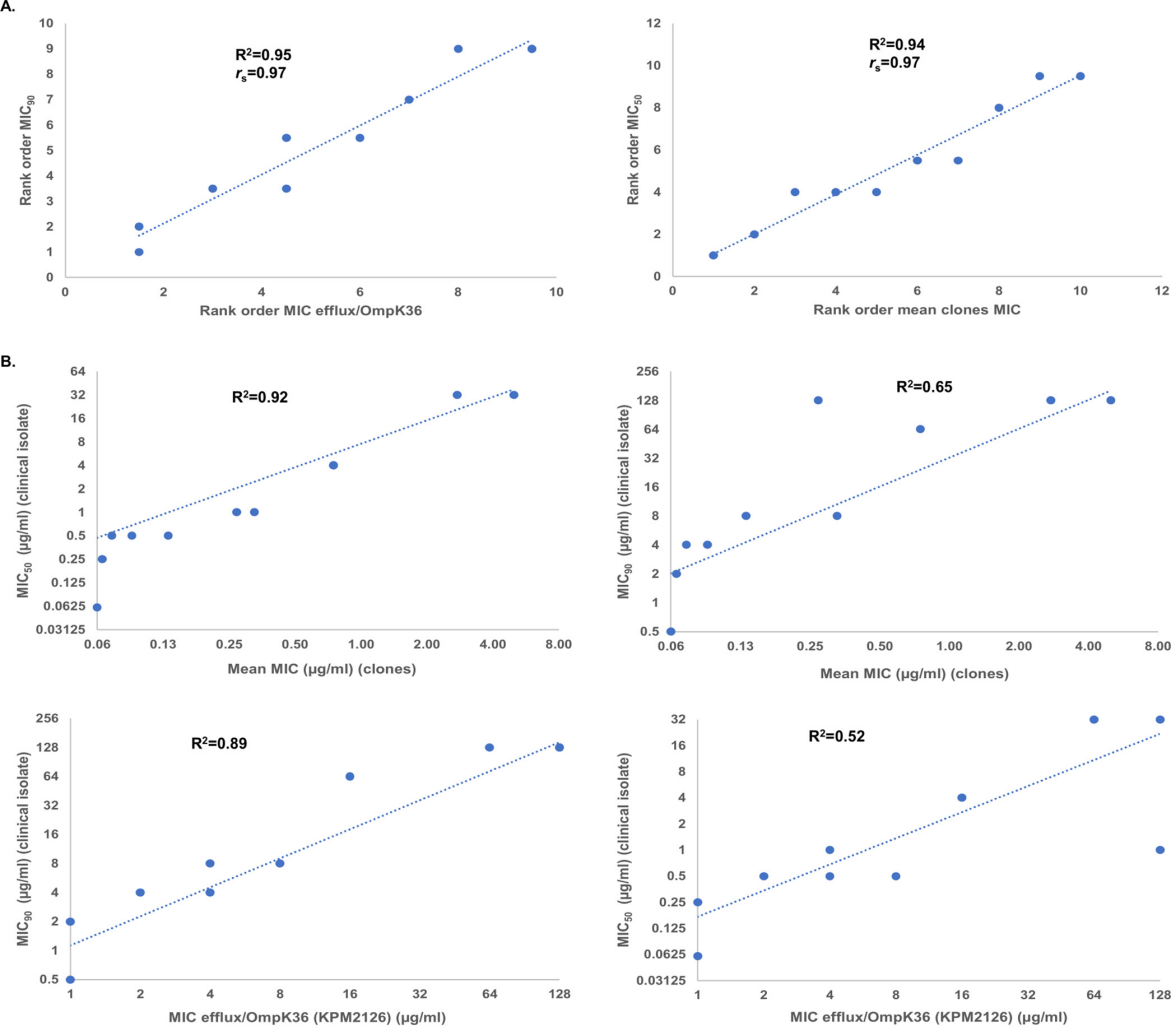
Evaluation of the correlations between potency of beta-lactams and their QPX7728 combinations against laboratory strains and clinical isolates. (A) Rank correlations; (B) linear correlations.

The impact of beta-lactamases and their inhibition by QPX7728 as well as general intrinsic/multidrug resistance (MDR) mechanisms on the observed potencies of various QPX7728 combinations against clinical isolates was evaluated using Spearman rank correlation. We ranked beta-lactam/QPX7728 combinations based on mean MIC against beta-lactamase-producing clones and by their MICs against isogenic mutants with various combinations of efflux and porin mutants (Table S2A). These rankings were compared with the rankings of the same combinations based on their MIC_50_s and MIC_90_s against clinical isolates. The Spearman correlation coefficients (*r*_s_) ranged from 0.82 to 0.97, indicating high correlation of both class-based and intrinsic MDR mechanisms (Table S2B). The highest correlation was observed between (i) the rank order based on the mean MICs of combinations against the panel of cloned beta-lactamases versus rank order based on their MIC_50_s against clinical isolates and (ii) the rank order based on MICs of beta-lactams alone against the strain that lacked both porins and had AcrAB-TolC overexpressed versus MIC_90_s of the corresponding combinations against clinical isolates: *r*_s_ was 0.97 (*P* value < 0.00001) (Fig. 3A).

In addition, we looked at linear correlation between the same variables. This analysis indicated that mean MICs of QPX7728 combinations versus clones were best correlated with MIC_50_s (*R*^2^ of 0.92 versus *R*^2^ of 0.65 for MIC_90_s). MICs of beta-lactams alone against the strain that possessed multiple intrinsic mechanisms of resistance were highly correlated with MIC_90_s of beta-lactam/QPX7728 combinations against clinical isolates (*R*^2^ of 0.89 versus *R*^2^ of 0.52 for MIC_50_) (Fig. 3B). Thus, the addition of QPX7728 to reverse beta-lactamase-mediated resistance for a beta-lactam antibiotic is necessary, but not sufficient, to achieve low MIC due to the effect of MDR/intrinsic resistance mechanisms on the beta-lactam.

## DISCUSSION

There has been considerable interest in developing new oral antibiotics to treat infections due to drug-resistant *Enterobacterales* in the outpatient setting. While oral fluroquinolones were used as first-line agents for treatment of many infections due to Gram-negative pathogens, increasing resistance as well as recognition of the safety issues around this class of drugs has reduced the utility of these agents. Oral beta-lactam antibiotics continue to have utility for treatment of some infections due to *Enterobacterales* (19–22), but their utility is similarly impacted by increasing resistance in ESBL- and carbapenemase-producing strains. Further, the effects of general intrinsic resistance mechanisms associated with multidrug resistance across antibiotic classes and the interplay between production of various beta-lactamases has not been systematically evaluated.

Most oral antibiotics were significantly impacted by efflux and porin mutations as assessed in an isogenic strain of K. pneumoniae. Tebipenem, ceftibuten, and cefixime were the agents least affected by these mechanisms, with only a 2- to 4-fold loss of activity in strains with single mutations in these mechanisms. However, when mutations impacting both permeability and efflux were present, all agents had a >8-fold loss in potency, with tebipenem being the most significantly affected, consistent with previous data on the effect of these mutations on other parenteral carbapenems.

This study confirmed that most oral beta-lactam antibiotics have low activity in *Enterobacterales* producing various extended-spectrum class A and class C beta-lactamases and serine beta-lactamases or metallocarbapenemases. Using the panel of isogenic strains with cloned beta-lactamases, it was shown that oral beta-lactams differed significantly in susceptibility to beta-lactamase-mediated hydrolysis. Overall, tebipenem and ceftibuten were the most stable to hydrolysis by common beta-lactamases present in *Enterobacterales*. We consider an increased enzymatic stability of a beta-lactam an advantage for use with any beta-lactamase inhibitor. More stable beta-lactams may be of greatest importance in the case of activity against strains that hyperproduce certain beta-lactamases or coproduce multiple beta-lactamases even in the presence of an inhibitor. In addition, the more stable the beta-lactam is as a substrate to a particular beta-lactamase, the lower is the concentration of the inhibitor required to fully restore the potency of the partner beta-lactam.

When tested in combination with QPX7728, there was a significant enhancement of the potency of most, but not all, oral beta-lactam antibiotics. These differences could be traced to both the susceptibility of each beta-lactam to beta-lactamase-mediated hydrolysis and impact of QPX7728 as well as susceptibility of the beta-lactam to general intrinsic mechanisms of resistance such as increased efflux and decreased uptake due to porin mutations. Using the panel of isogenic strains, the beta-lactams tebipenem, ceftibuten, and amdinocillin had the best response to testing with QPX7728; QPX7728 completely reversed beta-lactamase-mediated resistance irrespective of the produced beta-lactamase. Potentiation of other beta-lactams was generally less, particularly for strains producing MBLs such as NDM-1. The mechanisms underlying these differences in response are the subject of future studies, with the working hypothesis that these differences stem from the interplay between efficiency of enzymatic hydrolysis, inactivation efficiency, and efficiency of penicillin-binding protein (PBP) target inactivation by various beta-lactams. Complete reversal of resistance to a beta-lactam in the presence of the same amount of a BLI irrespective of the type of beta-lactamase represents an obvious advantage.

In the panel of isogenic strains with different efflux and porin mutations, ceftibuten, tebipenem, and cefixime had the lowest MICs against strains with multiple permeability defects owing to both intrinsic potency and reduced effect of efflux and porin-mediated resistance. As beta-lactam resistance due to porin and efflux mutations cannot be reversed by BLIs (23–25), including QPX7728 (18) combination products based on beta-lactams that are the least affected by these general mechanisms will have an obvious advantage.

In the panel of clinical isolates of beta-lactamase-producing strains of *Enterobacterales*, QPX7728 significantly increased the potency of oral beta-lactams across all enzyme subgroups. There was a high correlation between the ranking of potency of beta-lactam/QPX7728 combinations against beta-lactamase-producing clones (mean MIC) and against clinical isolates (MIC_50_/MIC_90_). Ranking of beta-lactams based on their potencies against the laboratory mutants with a combination of efflux and porin mutations was also highly correlated with potency of beta-lactam/QPX7728 combinations against clinical isolates. These data underscore the importance of overcoming both beta-lactamase-mediated and general intrinsic MDR mechanisms to achieve the highest potency against clinical isolates.

Based on overall microbiological profile described above, the carbapenem tebipenem and cephalosporin ceftibuten appeared to form the most potent combinations with QPX7728. Dosing, pharmacokinetic, pharmacokinetic-pharmacodynamic (PK-PD) relationships, and other pharmacologic considerations are additional factors that must be incorporated into choice of oral beta-lactams with QPX7728.

## MATERIALS AND METHODS

### Bacterial strains.

A set of isogenic strains of K. pneumoniae expressing individual cloned beta-lactamases was used to evaluate susceptibility of beta-lactams to beta-lactamase-mediated hydrolysis (14). The set of isogenic strains of K. pneumoniae with various combinations of efflux and porin mutations (18) was used in order to evaluate the impact of reduced uptake and increased efflux on susceptibility to beta-lactams alone. A total of 230 clinical isolates of *Enterobacterales* from the Qpex internal collection were used to evaluate the impact of QPX7728 on the potencies of various beta-lactams. These strains were selected to represent a broad cross-section of organisms, genotypes, and beta-lactamases. The panel included 47 Escherichia coli isolates, 139 Klebsiella pneumoniae isolates, 25 Enterobacter cloacae complex isolates, 4 Klebsiella oxytoca isolates, 5 Proteus mirabilis isolates, 4 Serratia marcescens isolates, 3 Citrobacter freundii isolates, 2 Enterobacter aerogenes isolates, and 1 Providencia stuartii isolate. Beta-lactamases were previously characterized in all the isolates, either by PCR followed by sequencing or by whole-genome sequencing. This information is summarized in Tables S1 and S2. Briefly, 84 strains produced various class A extended-spectrum beta-lactamases (ESBLs) with no carbapenemases or class C plasmidic enzymes (CTX-M, SHV, TEM, PER, and VEB), 48 strains produced class A carbapenemases (mainly KPC-2/KPC-3), 12 strains produced plasmidic class C beta-lactamases with no carbapenemases (mainly CMY), 48 strains produced OXA-48-like class D carbapenemases, and 36 strains produced class B metallo-beta-lactamases (19 NDM, 15 VIM, and 2 IMP). Of note, the majority of isolates coproduced two or more beta-lactamases; carbapenemases were often present together with class A ESBLs and/or class C enzymes.

### Antimicrobial susceptibility testing.

Bacterial isolates were subjected to broth microdilution susceptibility testing using panels prepared in-house and performed according to Clinical and Laboratory Standards Institute (CLSI) methods (26, 27). Cation-adjusted Mueller-Hinton broth (CAMHB; Becton, Dickinson, Sparks, MD) was used as a test medium. Tebipenem and ceftibuten were from BOC Sciences (Shirley, NY), cefixime, cefdinir, cefuroxime, cephalexin, cefditoren, and amdinocillin were from Sigma-Aldrich (St. Louis, MO), and cefpodoxime was from Carbosynth (San Diego, CA).

### Statistical analysis.

Spearman rank correlation coefficient was calculated using the CORREL Excel function.
