# Supplementary material for: The Ultrabroad-Spectrum Beta-Lactamase Inhibitor QPX7728 Restores the Potency of Multiple Oral Beta-Lactam Antibiotics against Beta-Lactamase-Producing Strains of Resistant Enterobacterales
Source: Antimicrob Agents Chemother. 2022 Feb 15;66(2):e02168-21. doi: 10.1128/aac.02168-21 (PMC8846479; doi:10.1128/aac.02168-21)
Supplement: Supplemental file 1 — Supplemental material. Download aac.02168-21-s0001.pdf, PDF file, 0.2 MB [file aac.02168-21-s0001.pdf]

SUPPLEMENTARY MARRIAL

SUPPLEMENTARY TABLES

Table S1. Acquired beta-lactamases identified in the strains used in this study[1]

	Molecular Class	*Escherichia coli*	*Klebsiella pneumoniae*	*Enterobacter cloacae species complex*	Others	Total
**CTX-M-2**	A	3	4			7
**CTX-M-3**	A		3	1	1	5
**CTX-M-14**	A	5	4	1		10
**CTX-M-15**	A	18	91	7	2	118
**CTX-M-27**	A	3				3
**CTX-M-55**	A	1			1	2
**SHV-2**	A	1				1
**SHV-26**	A		2			2
**SHV-28**	A		11			11
**SHV-12**	A	2	11		1	14
**SHV-18**	A		1			1
**SHV-40**	A				1	1
**TEM-1**	A	18	52	13	4	87
**TEM-12**	A	1				1
**TEM-116**	A	1				1
**TEM-135**	A	1				1
**TEM-2**	A				1	1
**TEM-26**	A	1	1			2
**TEM-28**	A		1			1
**TEM-30**	A	3	1			4
**TEM-35**	A	1				1
**TEM-40**	A	2				2
**PER-2**	A		1		1	2
**VEB-1**	A		1		1	2
**VEB-3**	A		2	2		4
**OXY-2-6**	A				2	2
**OXA-1**	D	12	30	5	1	48
**OXA-10**	D		4		1	5
**OXA-9**	D	1	15			16
**DHA-1**	C		3			3
**CMY-2**	C	6	1			7
**CMY-4**	C		5			5
**CMY-42**	C	1				1
**CMY-6**	C		1			1
**CMY-16**	C				1	1
**CMY-94**	C		1			1
**CMY-99**	C				1	1
**KPC[2]**	A, carbapenemase	6	25	12	5	48
**OXA-48-like[3]**	D, carbapenemase	3	41	1	3	48
**MBL[4]**	B	4	21	5	6	36

[1] Narrow spectrum class A chromosomal beta-lactamases (SHV-1/SHV-11/SHV-24 are not included). Many bacterial cells co-produced two or more beta-lactamases.
[2] KPC-2, 29; KPC-3, 14, KPC-4, 2; KPC-33, 1. Other class A carbapenemases: NMC-A, 1; SME-1, 1.
[3] OXA-48, 32; OXA-181, 8; OXA-232, 5; OXA-162, 2; OXA-163, 1.
[4] NDM-1, 16; NDM-4, 1; NDM-6, 2; VIM-1, 10; VIM-2, 2; VIM-27, 1; VIM-4, 1; VIM-5, 1; IMP-1,

Table S2. Spearman rank correlations

A. Rank orders of beta-lactams

Beta-lactam	WT MIC (KPM1026a) (µg/ml)	MIC Rank	MIC efflux (KPM1027) (µg/ml)	Efflux rank	OmpK36 MIC (KPM2040) (µg/ml)	OmpK36 rank)	OmpK35 OmpK36 MIC (KPM2613) (µg/ml)	OmpK35 OmpK36 rank	MIC OmpK36/ efflux (KPM2126) (µg/ml)	Efflux/OmpK36 rank	Mean clones MIC (µg/ml)	Mean clones rank	MIC_{50} (µg/ml)	MIC_{50} rank	MIC_{90} (µg/ml)	MIC_{90} rank
Cefaclor	0.5	**7**	1	**4.5**	2	**6.5**	4	**7**	16	**7**	0.75	**8**	4	**8**	64	**7**
Cefalexin	4	**10**	8	**9**	16	**9**	32	**9**	128	**9.5**	5.00	**10**	32	**9.5**	128	**9**
Cefdinir	0.125	**3.5**	1	**4.5**	2	**6.5**	2	**6**	8	**6**	0.13	**5**	0.5	**4**	8	**5.5**
Cefditoren	0.5	**7**	2	**7**	1	**5**	1	**4.5**	4	**4.5**	0.07	**3**	0.5	**4**	4	**3.5**
Cefixime	0.125	**3.5**	0.5	**3**	0.25	**2.5**	0.5	**2.5**	2	**3**	0.09	**4**	0.5	**4**	4	**3.5**
Cefpodoxime	0.125	**3.5**	2	**7**	0.5	**4**	1	**4.5**	4	**4.5**	0.33	**7**	1	**5.5**	8	**5.5**
Ceftibuten	0.125	**3.5**	0.25	**2**	0.25	**2.5**	0.25	**1**	1	**1.5**	0.07	**2**	0.25	**2**	2	**2**
Cefuroxime	2	**9**	32	**10**	16	**9**	16	**8**	64	**8**	2.75	**9**	32	**9.5**	128	**9**
Mecillinam	0.5	**7**	2	**7**	16	**9**	128	**10**	128	**9.5**	0.27	**6**	1	**5.5**	128	**9**
Tebipenem	0.06	**1**	≤0.06	**1**	0.125	**1**	0.5	**2.5**	1	**1.5**	0.06	**1**	0.06	**1**	0.5	**1**

B. Spearman correlation coefficients

	MIC_{50} rank	P_{value}	MIC_{90} rank	P_{value}
**Mean MIC clones rank**	0.97	0.000003	0.91	0.0002
**wild type MIC rank (based on KPM1026a MIC)**	0.87	0.001	0.82	0.003
**Efflux rank (based on KP1027 MIC)**	0.83	0.003	0.83	0.003
**OmpK36 rank (based on KPM2040 MIC)**	0.83	0.003	0.96	0.00002
**OmpK35 OmpK36 rank (based on KPM2613 MIC)**	0.79	0.007	0.95	0.00003
**OmpK36/efflux rank (based on KPM2126 MIC)**	0.84	0.002	0.97	0.000003
